# Electrochemical Applications Reveal Enhanced Photocatalytic Performance of TiO_2_ ‐Doped ZnS Nanocomposites

**DOI:** 10.1002/jemt.24722

**Published:** 2024-10-30

**Authors:** S. Synthiya, T. Thilagavathi, R. Uthrakumar, R. Renuka, C. Inmozhi, K. Kaviyarasu

**Affiliations:** ^1^ Department of Physics Government College for Women (Autonomous) Kumbakonam Tamil Nadu India; ^2^ Affiliated to Bharathidasan University Tiruchirappalli Tamil Nadu India; ^3^ Department of Physics Government Arts College (Autonomous) Salem Tamil Nadu India; ^4^ Department of Physics Government Arts College for Women (Autonomous) Pudukkottai Tamil Nadu India; ^5^ Department of Physics Govt. Arts College for Women Salem Tamil Nadu India; ^6^ UNESCO–UNISA Africa Chair in Nanosciences/Nanotechnology Laboratories, College of Graduate Studies University of South Africa (UNISA) Pretoria South Africa

**Keywords:** environmental pollution, photodegradation procedures, photoelectrochemical, *pseudo‐first‐order* kinetics, TiO_2_‐doped ZnS nanocomposites

## Abstract

The titanium dioxide (TiO_2_) nanoparticles were prepared by hydrothermal methods at ambient temperature. Based on XRD analysis, the average crystallite size of pure TiO_2_ nanoparticles and those doped with ZnS was calculated to be 58 and 54 nm, respectively. At an angle of 25.4°, the prominent peak observed at the (101) plane of TiO_2_ was confirmed. As can be seen from the collection of peaks, the TiO_2_ formed has an anatase‐type tetragonal crystal structure. A strain of −6.4541 × 10^−4^ and a grain size of 33 nm can be seen in the W–H plot for TiO_2_ nanoparticles. For doped ZnS nanoparticles, on the other hand, the values are 1.9448 × 10^−4^ and 47 nm. In our study, we found that doped nanoparticles were average grain size 134 nm, while pure nanoparticles were average grain size 146 nm. Doping reduces the size of the nanomaterial, which means that the TiO_2_ molecules form nanoclusters on their surfaces, which can lead to a larger grain size for a pure nanoparticle than for a doped nanoparticle. A wide range of functional groups and their associated bonds were investigated using FTIR spectra in synthesized nanomaterials. Ti—O—Ti bonds are subjected to a strong stretching vibration, which is confirmed by the absorption peaks from 450 cm^−1^ to 800 cm^−1^. The PL spectra for pure TiO_2_‐ and ZnS‐doped TiO_2_ containing nanocomposites of ZnS emit ultraviolet light at wavelengths of 362 and 379 nm in the UV region. Pure and doped samples with optical bandgap energies of ~3.04 and ~3.8 eV corresponding to anatase phases were near ~3.18 eV in *Tauc plots*. Since the TiO_2_‐doped ZnS heterojunction migrates photoexcited holes toward the interface, while electrons migrate toward the bulk, this results in photoexcited holes migrating toward the interface. To calculate the specific capacitance of the synthesized materials, cyclic voltammetry with pure ZnS and those with ZnS‐doped had specific capacitance values of 144.91 F/g and 120.11 F/g, respectively. The catalysts used were ZnS nanocomposite doped with TiO_2_ in addition to pure TiO_2_ nanoparticles. The degradation of dye within 80 min after sunlight exposure was monitored with a UV–Vis spectrophotometer at 20‐min intervals. ZnS nanoparticles doped with TiO_2_ display 87.8% greater efficiency than pure nanoparticles. Doped TiO_2_ nanocomposite degrades at an 87.8% rate, whereas pure TiO_2_ degrades at ~54%, indicating that the dopants enhance photocatalysis.

## Graphical abstract

TiO_2_ nanoparticles were synthesized using low‐temperature hydrothermal methods. A 1 M TiO_2_ sample was mixed with 50 mL of CH_3_COOH in a 500‐mL beaker using TiO_2_ as a precursor. The solution was then stirred at room temperature using a Remi digital high‐speed stirrer. Following the addition of 50 mL of distilled water, the solution was stirred for an hour. The pH level of the mixture was regulated by adding NaOH solution. In the process of dye degradation, first‐order kinetics is involved. The absorption values of ZnS nanoparticles and TiO_2_‐doped ZnS nanocomposites were determined by analyzing UV–Vis spectra at different time points. At the initial time point (*t* = 0), 617 nm was observed to be the point of maximum absorption. Dopants cause slight shifts in maximum wavelengths due to their presence. In the presence of visible light, pure TiO_2_ and TiO_2_‐doped ZnS nanocomposites are shown to be able to degrade MG dye through a photocatalytic mechanism. After 80 min, pure ZnS nanocomposites and TiO_2_‐doped ZnS nanocomposites degraded aqueous samples with degradation efficiencies of ~54% and ~88%, respectively, demonstrating their photocatalytic capabilities.

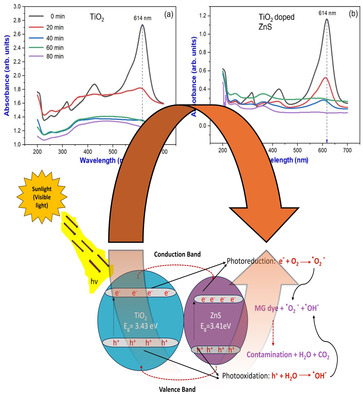

**Summary**

- Average crystallite size of pure TiO_2_ NPs and doped ZnS was 58 and 54 nm.
- On the other hand, the values for doped TiO_2_ nanoparticles are –1.9448 × 10^–4^ and 47.811 nm.
- Stretching vibration associated with Ti‐O‐Ti bonds as shown by the absorption peaks from 450 to 800 cm^–1^.
- TiO_2_ doped ZnS nanocomposites emit UV light at 363.07 and 377 nm, violet light at 411.05 nm, and blue light at 490 nm.
- *Tauc plot* gave optical bandgap energy of 3.04 and 3.8 eV for both pure and doped samples which were nearly in *anatase phase* of TiO_2_ which is 3.18 eV.
- Maximum absorption was observed at 614 nm at the initial time point (*t* = 0). Due to the presence of the dopant, there is a slight shift in the maximum wavelength.

## 1 Introduction

Due to their unique properties such as smaller sizes, novel properties, and high surface‐to‐volume (*s*/*v*) ratios, nanomaterials have been used in a wide variety of industries, such as chemical, electronic, food, textile, and agriculture, in place of traditional bulk materials (Kumari, Alam, and Ahmed Siddiqi 2019; Ali Yaqoob et al. 2020). Nanoscale metal oxides, such as titanium dioxide (TiO_2_), have been highly regarded for their potential applications in numerous fields, including wastewater treatment (Akakuru, Iqbal, and Aiguo 2020; Choi, Jung, and Kim 2005). In recent years, TiO_2_ nanoparticles have been synthesized based on the increased *s*/*v* ratios they exhibit as their size decreases (Chen and Mao 2007). The TiO_2_ nanoparticles exhibit different chemical and physical properties for different crystal structures. There are three kinds of phases in this class: anatase, rutile, and brookite. The rutile and anatase phases are the most common, and they have tetragonal shapes, while brookite is orthorhombic (Fujishima and Honda 1972; Alivisatos 1996; Barnard, Erdin, and Lin 2006). As a result of their optical bandgaps of 3.0 and 3.2 eV, the rutile and anatase phases are well‐suited for optoelectronic applications. As a result of its stability, anatase is commonly used in solar cells (Ahmari, Heris, and Khayyat 2018). For these unique characteristics, TiO_2_ nanoparticles are indispensable in paint, paper, pesticide removal, soil remediation, food, cosmetics, hydrogen production, energy devices, wastewater treatment, nanomedicine, nanobiotechnology, and other industries. To synthesize TiO_2_ nanoparticles, different techniques were employed. In addition to physical vapor deposition (PVD) and chemical vapor deposition (CVD), oxidation and solvothermal methods, sol–gel methods, thermal decomposition, hydrothermal techniques, and biological extraction using plants and microorganisms are also used.

As a result of their cost‐effectiveness, efficiency, and ease of use, chemical methods were preferred. It has been demonstrated in one study that the hydrothermal synthesis method can be used to produce both pure TiO_2_ nanoparticles and TiO_2_‐doped zinc sulfide (ZnS) nanoparticles at low temperatures. Using these syntheses, the main objective was to investigate their photocatalytic properties (Irshad et al. 2021; Shahat et al. 2021). It was previously demonstrated that TiO_2_ nanoparticles are capable of breaking down harmful substances, including toxic azo dyes, which are found in wastewater and in the air (Phattepur, Bychapur Siddaiah, and Ganganagappa 2019; Gopinath et al. 2020). To assess the efficacy of TiO_2_ nanoparticles in treating wastewater, numerous experiments have been conducted. The photocatalytic activity of pure TiO_2_ and TiO_2_‐doped ZnS nanoparticles was investigated under sunlight conditions using malachite green (MG) dye (Kubiak et al. 2020). A study with the aim of determining dye degradation rate constants and percentage efficiency was conducted (Wadhwa et al. 2020; Jang et al. 2006).

Despite the advantages of titania as a photocatalyst, its applications are limited because of its low bandgap and high recombination rate of charge carriers. There has been a great deal of concern among researchers regarding these issues. A recent method to improve TiO_2_'s photocatalytic capability has been to modify the electronic structure through doping with a suitable dopant. The ability to absorb visible light has been demonstrated by noble metals when doped into semiconductor lattices. As well, a Schottky barrier separates electron‐hole pairs (e^−^/h^+^) at the interface between the semiconductor and noble metal. Due to its affordability, natural optoelectric properties, and ease of preparation, ZnS is one of the most promising metals for this purpose. Our research involved the synthesis and characterization of nanosized TiO_2_ doped with ZnS, and to study their optical and structural properties. As a result of sunlight exposure, their photocatalytic performance was evaluated for the degradation of MG, which was reported in detail.

## 2 Methodology

### 2.1 Chemicals and Reagents

We purchased TiO_2_, ZnS, and sodium hydroxide pellets (NaOH) from Sigma–Aldrich in their analytical reagent grade without further purification. This experiment was conducted with distilled water as the solvent.

### 2.2 Synthesis of Pure TiO_2_ and TiO_2_ ‐Doped ZnS Nanocomposites

**FIGURE 1:**
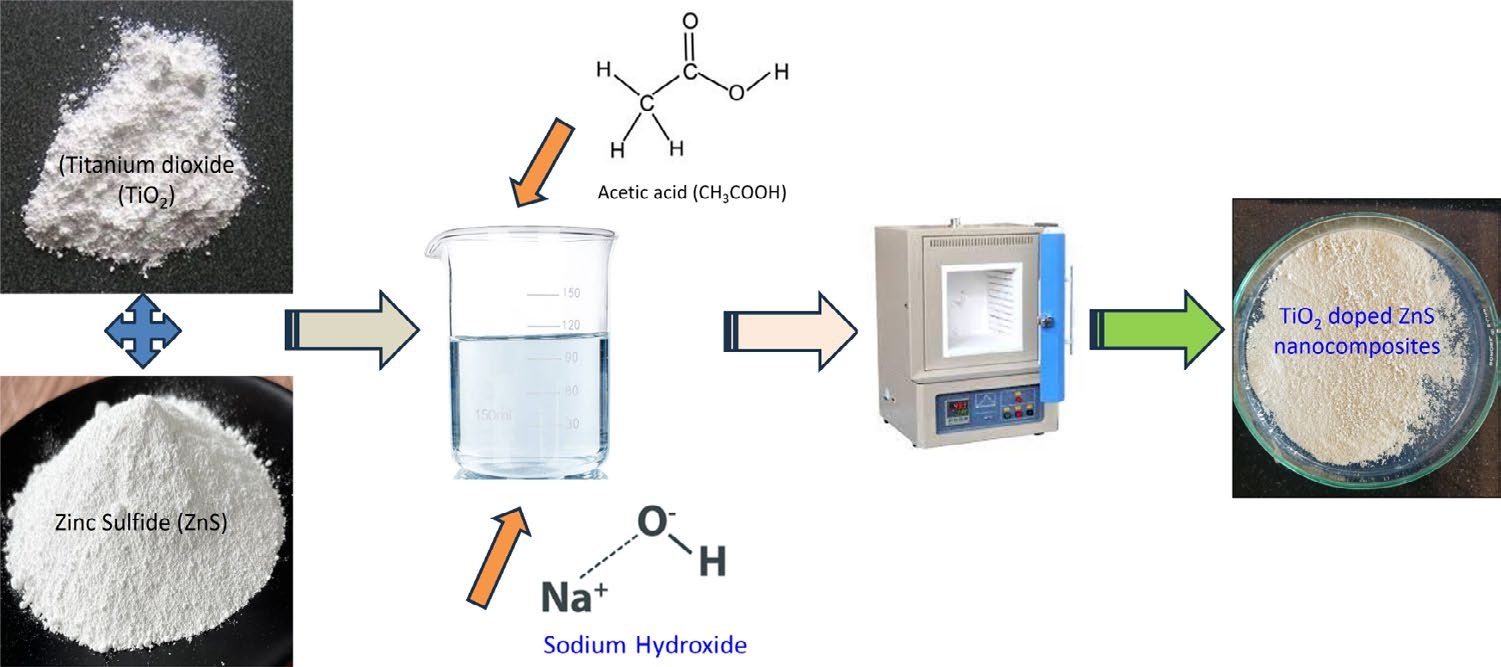
An illustration of the methodology for preparing TiO_2_‐doped ZnS nanocomposites.

The solution of TiO_2_ and ZnS, dissolved in 100 mL of distilled water, was agitated vigorously and maintained at ~8.2 pH by added sodium hydroxide solution. The solution containing TiO_2_ doped ZnS was vigorously stirred before adding sodium hydroxide solution drop by drop. As the solution precipitated, it developed a whitish color within 5 min. As part of the reaction, the solution was heated at 150°C for 9 h in a Teflon‐lined autoclave. An annealing process of 7 h at 530°C was used to anneal the synthesized nanoparticles. After the reaction was complete, the furnace took a few minutes to cool down to room temperature. The solid products were then washed with absolute ethanol and distilled water. A 3‐h drying period at 110°C was followed by a cooling process. Based on the schematic representation of the samples in Figure 1, a variety of characterization techniques were applied to analyze and understand the TiO_2_‐doped ZnS nanocomposites.

## 3 Results and Discussion

### 3.1 Powder X‐Ray Diffraction

**FIGURE 2:**
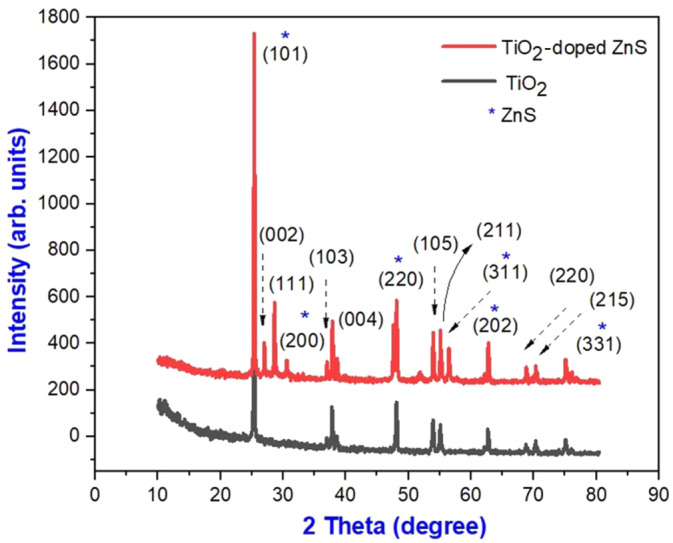
XRD pattern of pure TiO_2_ and TiO_2_‐doped ZnS nanocomposites. * is the ZnS symbol.

TiO_2_ nanoparticles in the anatase phase exhibit peaks like those observed in the XRD pattern as shown in Figure 2. The most prominent peaks are observed at plane (101) and others at planes (004), (200), (220), (311), and (331). It is likely that the ZnS nanoparticles were responsible for causing the peaks to be shifted when TiO_2_ was doped with ZnS nanoparticles. In accordance with (Negi et al. 2021) the Debye–Scherrer formula (1), the average crystallite size of the nanoparticles was determined. In addition to analyzing crystalline phases of materials and determining lattice parameters, crystal systems, strains, dislocation densities, epitaxy, phase compositions, orientations, and defects, x‐ray diffraction (XRD) is an effective tool for analyzing structural properties.

(1) $$D=\frac{\mathrm{k\lambda}}{\beta \;\cos\theta }$$

where “*D*” is the crystallite size, “*λ*” is the wavelength of the x‐rays = 1.5405 Å, “*k*” is the particle factor = 0.94, “*β*” is the full‐width half‐maximum, and “*θ*” is Bragg's diffraction angle. The average crystallite size of pure TiO_2_ nanoparticles and those doped with ZnS was 58 and 54 nm, respectively. The prominent peak observed at an angle of 2*θ* = 25.4° indicates the presence of the (101) plane of TiO_2_. It is confirmed by the collection of peaks that the TiO_2_ prepared has a tetragonal crystal structure that is of the anatase type. Furthermore, some rutile peaks at 27.5° and 37.2°, which represent the (111) and (103) planes, were also identified in the doped nanoparticles. As a result, the sample seems to have both anatase and *rutile* phases. It has a large surface area, which means there are more active sites, making it more reactive. It could be explained by this reason that the stable rutile phase is converted into the reactive anatase phase. There were no additional peaks indicating impurities, which was an important finding. Because of the high degree of crystallinity in the diffraction peaks, carriers of charge are more likely to reach the surface when trapped by crystal defects. Moreover, the particles are smaller, so they travel a shorter distance before reaching the surface, reducing the chances of recombination. This increased efficiency in charge carrier transport has a major effect on the material's reactivity and performance.

To obtain an accurate value of the average crystallite size of the nanoparticles, we used the modified Debye Scherrer method, which consists of the following equations:

(2) $$\ln\;\beta =\ln\;\frac{1}{\cos\theta }+\ln\;\frac{\mathrm{k\lambda}}{D}$$

A crystallite's average size was calculated using updated equations to be 36 and 49 nm. Using Equations (4) and (5), we determined dislocation density and microstrain.

(3) $$\delta =\frac{1}{D^{2}}\;\left(\text{lines}\;m^{-2}\right)$$

(4) $$\varepsilon =\frac{\beta }{4\sin\theta }$$

By considering the full‐width half‐maximum and total broadening, the Williamson–Hall (W–H) method can determine the average size of nanoparticles and microstrains (Fan et al. 2020). This formula can be expressed as follows:

(5) $$\beta_{T}\cos\theta =\mathrm{k\lambda D}+4\varepsilon \;\sin\theta$$

**FIGURE 3:**
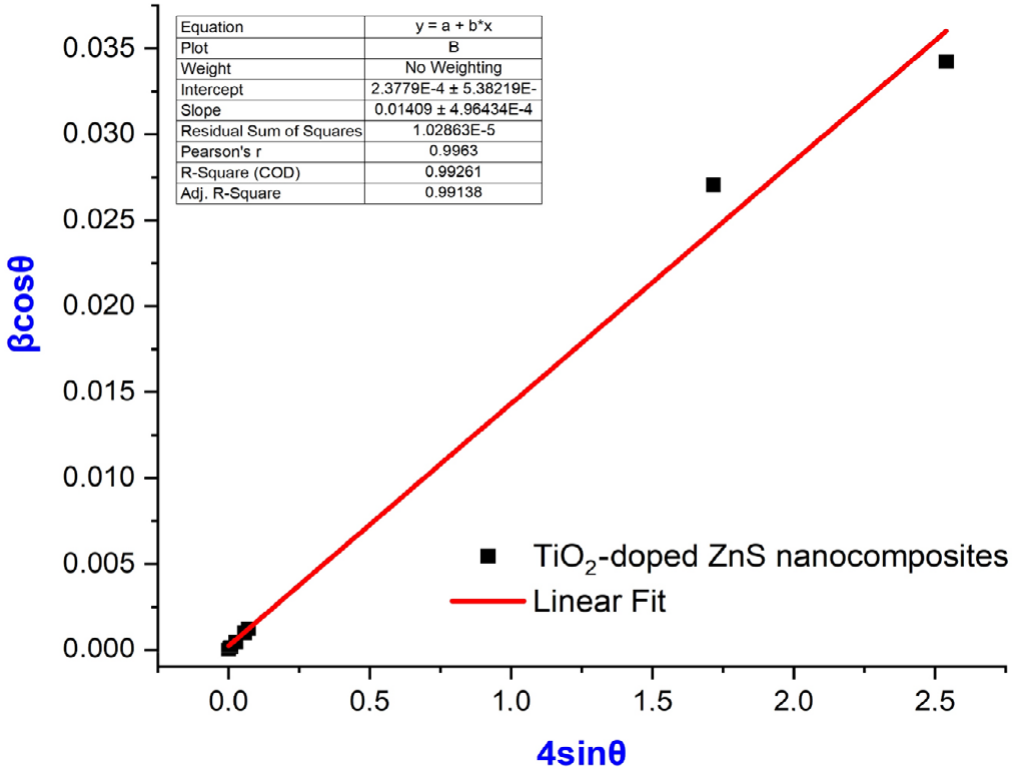
W–H plot of TiO_2_‐doped ZnS nanocomposite.

A plot of W–H as shown in Figure 3, TiO_2_ nanoparticles have a strain of −6.4541 × 10^−4^ and a grain size of 33 nm. On the other hand, the values for doped TiO_2_ nanoparticles are −1.9448 × 10^−4^ and 47 nm. Based on the crystallinity measurements, pure TiO_2_ nanoparticles showed a value of 21 nm, and TiO_2_‐doped ZnS nanocomposites showed a value of 40 nm. In other words, the crystallinity of these materials increased when they were doped with TiO_2_.

### 3.2 SEM Analysis and Electron‐Dispersive X‐Ray Spectroscopy

**FIGURE 4:**
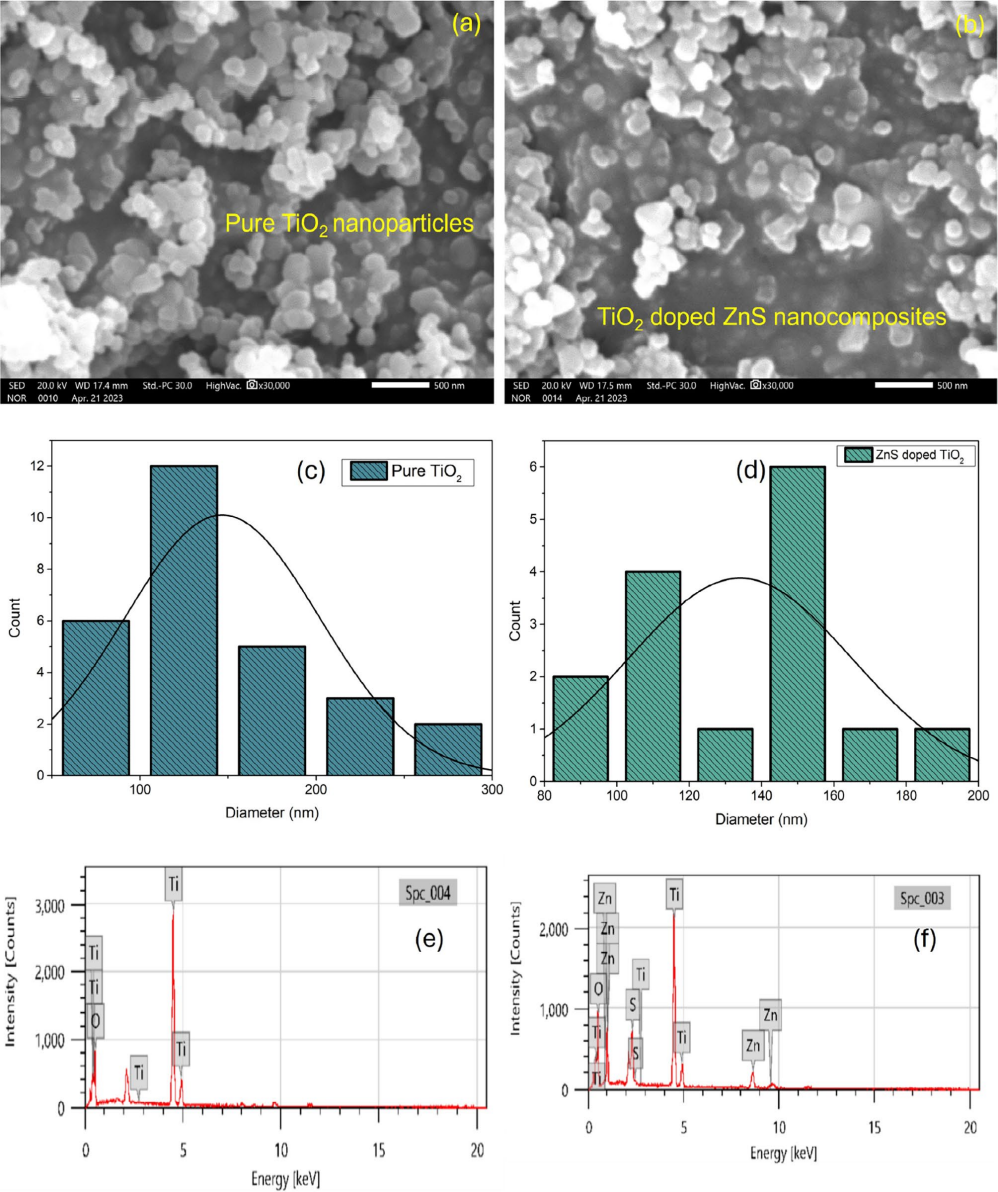
(a–f). SEM images, particle size distribution plot and EDS images of pure TiO_2_ nanoparticles, and TiO_2_‐doped ZnS nanocomposites.

Using scanning electron microscopy (SEM), the materials' structural characteristics were analyzed. A high‐magnification camera captured detailed images of the elements' surfaces. The SEM image of pure TiO_2_ nanoparticles and TiO_2_ nanoparticles doped with ZnS were shown in Figure 4a,b at a magnification of 500 nm. In addition to the irregular morphology of TiO_2_, the porous structure of the material allows ions to diffuse easily across electrodes, resulting in a high capacitance. The increase in porosity of the composite material will lead to higher photocatalytic performance (Fan et al. 2020). We found that the doped nanoparticles had an average grain size of 134 nm and pure nanoparticles had an average grain size of 146 nm. Compared to the doped nanoparticle, the pure nanoparticle has a larger grain size, suggesting that doping results in reduction in the size of the nanomaterial. Nanoclusters were formed on the surface of the TiO_2_ molecules. Based on the surface morphological investigation, a Gaussian fit is used to calculate the particle size distribution (Listani et al. 2019) in Figure 4c,d for the nanoparticles present on the surface. The average particle size of the TiO_2_ nanoparticles and TiO_2_‐doped ZnS nanocomposite were measured to be 124 and 151 nm, respectively. These measurements were not significantly different from the average particle size calculation using ImageJ software. To determine the elemental composition of various materials, energy‐dispersive x‐ray spectroscopy (EDS) is an effective analytical technique. As a result of EDS analysis of the sample, a series of peaks is displayed showing the presence and proportions of different elements. A schematic depiction of the atomic composition of the synthesized nanomaterials as shown in Figure 4e,f. Oxygen (O) and titanium (Ti) are the peaks in image (e), showing a pure form with no impurities; O accounts for 69.79% of the composition, while Ti makes for 30.21%. The TiO_2_‐doped ZnS nanocomposites have atomic percentages of 64.10% O, 24.04% Ti, 6.68% Zn, and 5.19% S without any additional components (Listani et al. 2019). From this, we conclude that both synthesized nanoparticles were in pure form without any accumulation.

### 3.3 FTIR Spectroscopy

**FIGURE 5:**
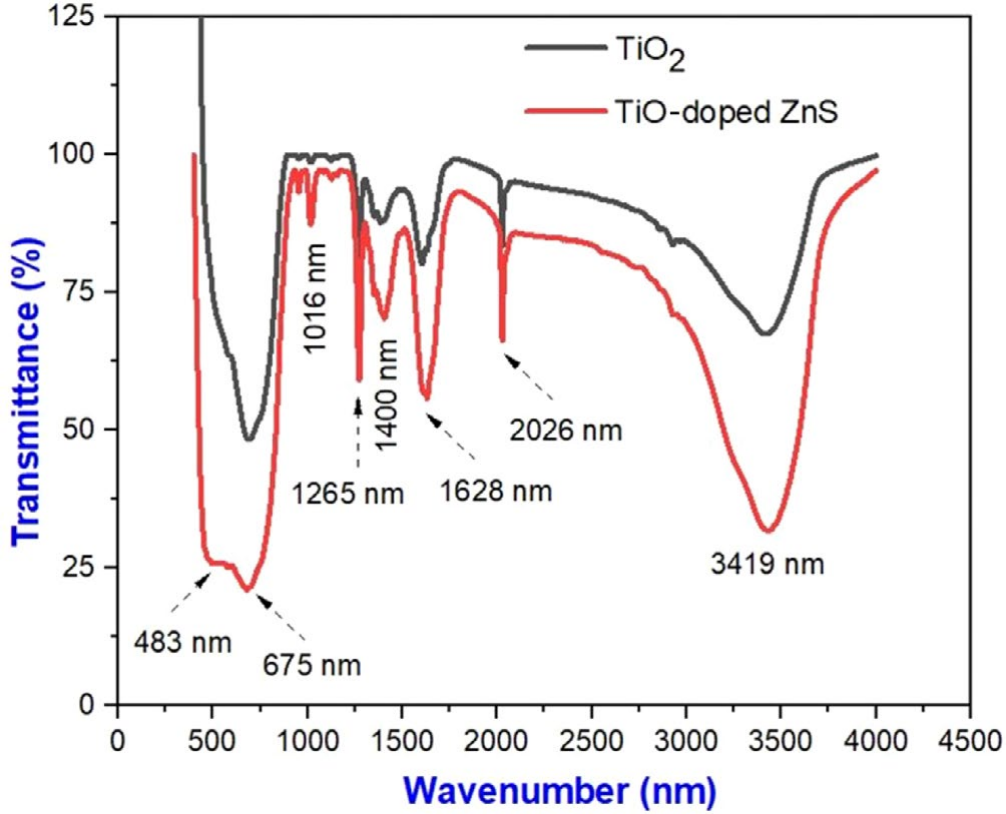
FTIR spectrum of pure TiO_2_ and TiO_2_‐doped ZnS nanocomposites.

Analyzing chemical bonds within materials using FTIR spectroscopy is an effective method without damaging them. It is possible to analyze materials qualitatively and quantitatively with FTIR spectra and determine whether they contain organic or functional groups (Thongpool et al. 2020). As shown in Figure 5, FTIR spectra were used to investigate various functional groups and their corresponding bonds in synthesized nanomaterials. As seen in Figure 5, there is a strong stretching vibration associated with Ti—O—Ti bonds as shown by the absorption peaks from 450 to 800 cm^−1^. Ti—O—Ti bending modes are represented by peaks between 1500 and 1700 cm^−1^, while Ti—O—Ti vibrational modes are presented by peaks between 1300 and 1400 cm^−1^ (Sun et al. 2008; Mirabedini et al. 2011). There is a signal at 1628 cm^−1^ that indicates the presence of aromatic C=C resonance groups. At 2926.55 cm^−1^, aliphatic C—H compounds with atomic strain vibration were detected. A stretching of O—H was observed between 3300 and 3600 cm^−1^ as a result of moisture adsorption. There appears to be an interaction between hydroxyl functional groups on TiO_2_ nanoparticle surfaces and ZnS functional groups, thereby reducing TiO_2_ nanoparticles. TiO_2_‐doped ZnS shows that phenolic compounds and other components play an important role in the formation and stabilization of TiO_2_ nanoparticles. It has been confirmed that metal‐oxygen bonds have been formed and are exhibited in TiO_2_ and TiO_2_‐doped ZnS spectra.

### 3.4 Photoluminescence Studies

**FIGURE 6:**
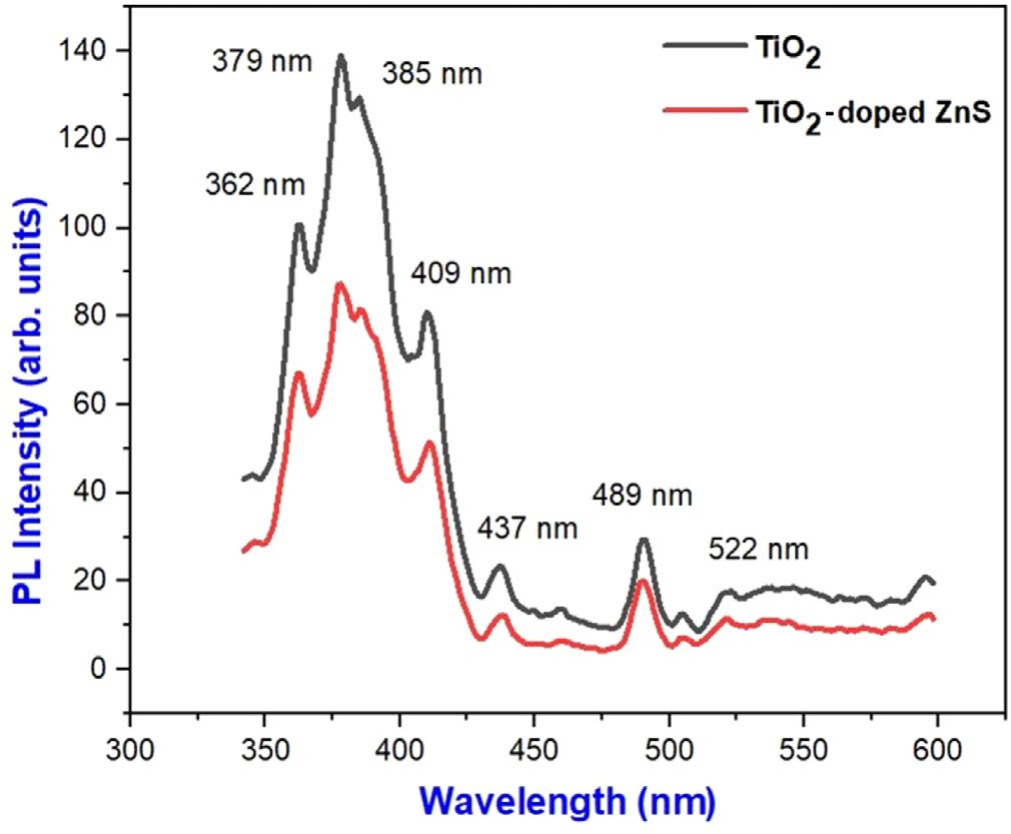
PL emission spectrum of pure TiO_2_ and TiO_2_‐doped ZnS nanocomposites.

To examine the differences between pure samples and doped samples, we conducted photoluminescence analyses. By absorbing light, electrons in the valence band (VB) become energetic and transition to the conduction band (CB). This results in electron–hole (e^−^/h^+^) pairs being formed as shown in Figure 6. In the visible spectrum, light is emitted by the interaction between these pairs. It is a characteristic of semiconductor materials to exhibit this phenomenon. It can be used to identify the structure, correlation, bandgap energy, and impurities or defects of a material by analyzing its photoluminescence (PL) spectrum. Here is an illustration of the PL spectrum for pure TiO_2_ compared with TiO_2_ containing nanocomposites of ZnS. The emission occurred in the UV region, at the wavelengths of 362 and 379 nm by TiO_2_ nanoparticles are not visible to the naked eye. In addition to emitting violet light at 409 nm, they also emit blue light at 489 nm. TiO_2_‐doped ZnS nanocomposites emit UV light at 363 nm and 377 nm, violet light at 411 nm, and blue light at 490 nm, respectively. The presence of dopants causes the shift in the peak in the doped sample. As shown in Table 1, the bandgap energy was determined from the PL data. Anatase phase can be clearly confirmed by the bandgap range of the synthesized TiO_2_ nanoparticles. As a result, doped nanoparticles will have greater photocatalytic activities than pure nanoparticles due to their slight decrease in bandgap energy, given in the Table 1.

**TABLE 1:** A comparison of the bandgap energy of pure TiO_2_ and TiO_2_‐doped ZnS nanocomposites and their emission spectra.

Sample	Optical bandgap energy, *E*_*g*_ (eV)	Emission wavelength, *Λ* (nm)	Color of emission
TiO_2_ (pure)	3.43 eV	409 and 489 nm	Violet, blue
TiO_2_‐doped ZnS nanocomposites	3.41 eV	411.05 and 490 nm	Violet, blue

### 3.5 UV–Vis Spectroscopy

**FIGURE 7:**
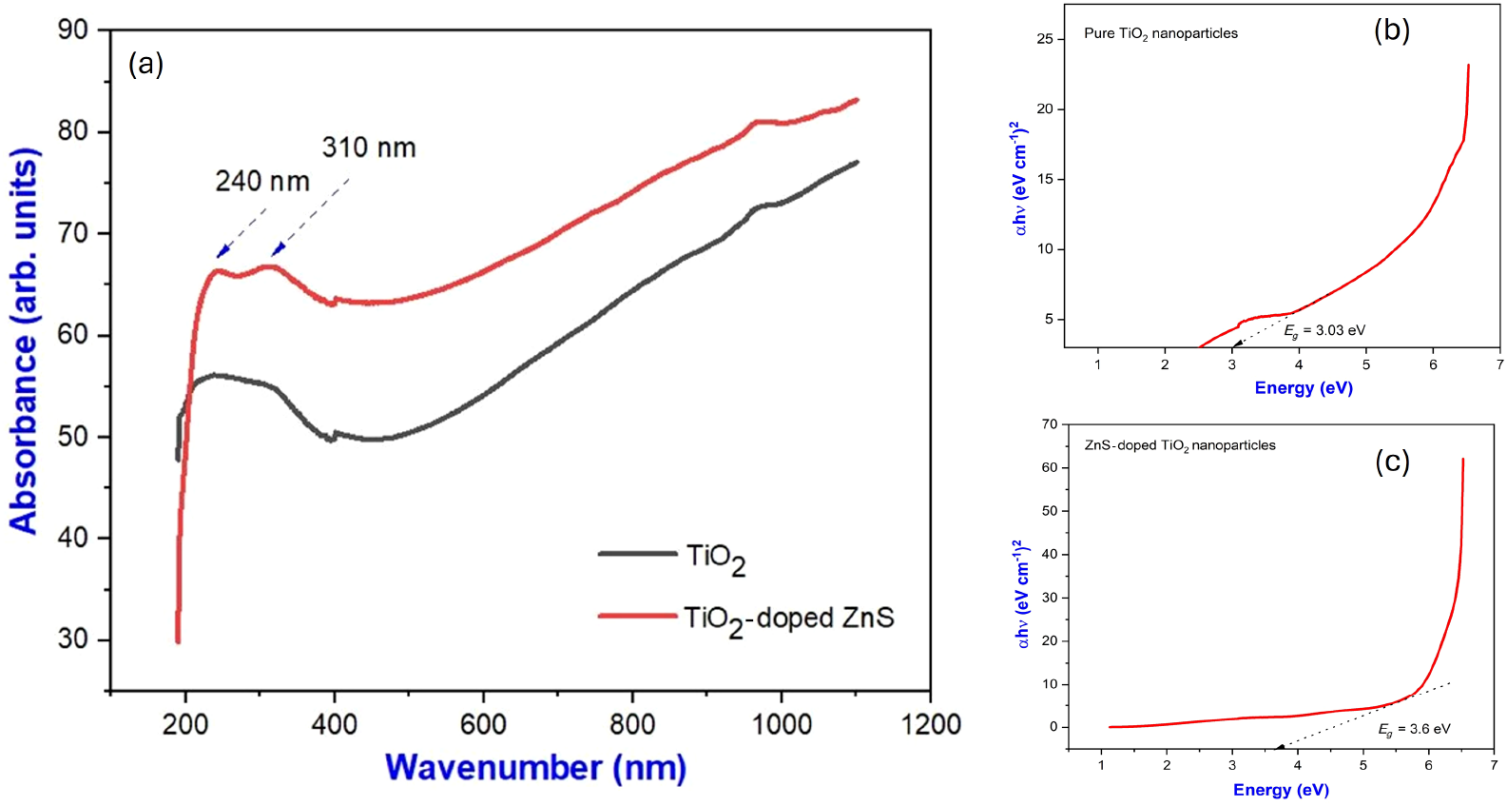
UV–Vis spectrum and *Tauc Plot* of pure TiO_2_ and TiO_2_‐doped ZnS nanocomposites.

Optical characteristics of the materials were examined using UV–visible spectroscopy. Based on these spectra, various factors would be identified, such as the energy of the optical bandgap, the size distribution, the shape, and the concentration of the materials. As a result of the optical bandgap energy, photocatalytic properties can be determined and materials classified as conducting, semiconducting, or superconducting materials. A UV–visible absorption spectrum ranging in wavelength from 100 to 1200 nm can be seen in Figure 7a–c. There is a strong absorption band in the 387 nm wavelength region of the absorption spectrum of the TiO_2_ lattice. Both components of TiO_2_‐doped ZnS exhibit two‐phase absorption bands. Around 700–750 nm, an additional broad peak form due to the presence of ZnS. By using the *Tauc Plot* method (Wu, Yu, and Fu 2006), we were able to measure the optical bandgap.

According to the Tauc Equation (6),

(6) $$\mathrm{\alpha h\nu}=A\;{\left(\mathrm{h\nu}-E_{g}\right)}^{n}$$

where “*α*” is the absorption coefficient, “*h*” is the Planck constant, “*ν*” is the frequency, “*A*” is the energy‐dependent constant, *E*_*g*_ is the optical bandgap energy of the material, and “*n*” is the nature of transition which will take different values *n* = 1/2, 3/2, 2, 3 for direct transition, forbidden direct transition, indirect transition, and forbidden indirect transitions, respectively. Here, TiO_2_ nanoparticles have a direct transition, so *n* = 1/2. The *Tauc plot* gave optical bandgap energy of 3.04 and 3.8 eV for both pure and doped samples which were nearly in the anatase phase of TiO_2_ which is 3.18 eV. It is clear from this bandgap range that chemically synthesized nanomaterials are semiconductors (Sun et al. 2008).

### 3.6 Electrochemical Studies

**FIGURE 8:**
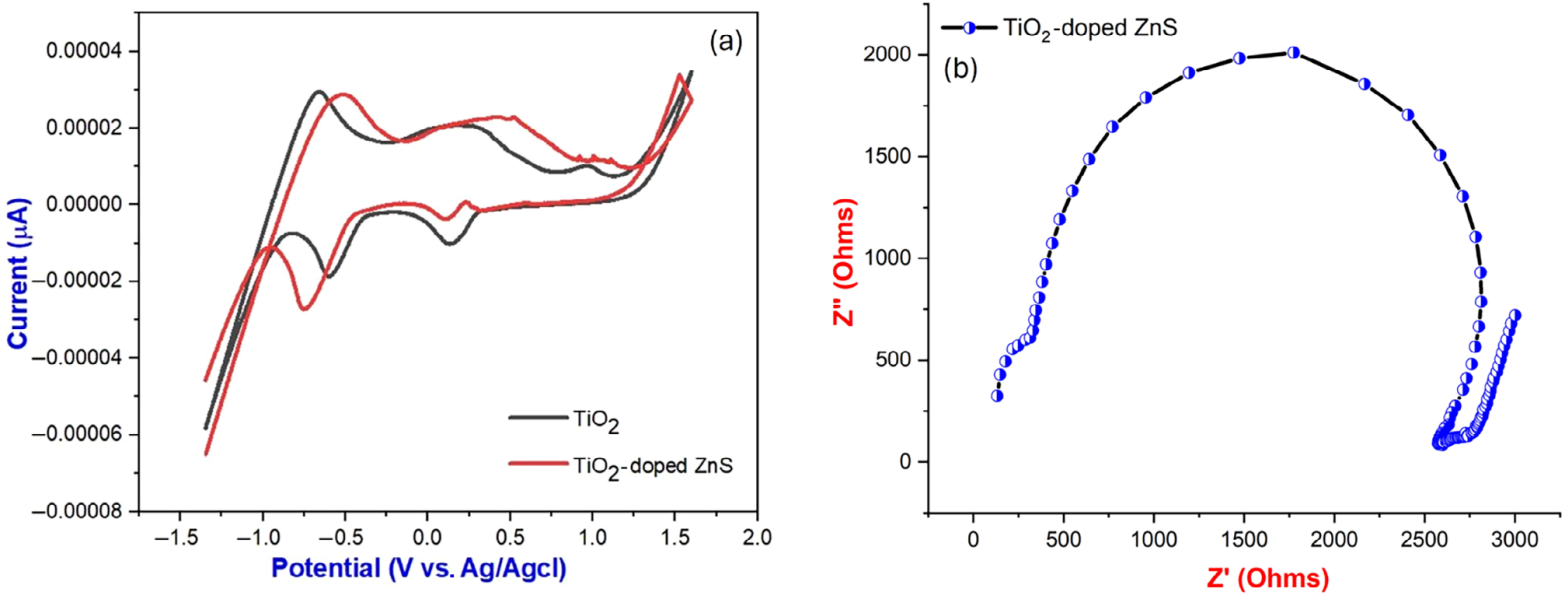
(a) Comparison of the CV curves for pure TiO_2_ and TiO_2_‐doped ZnS nanocomposites; and (b) Nyquist plots from electrochemical impedance spectroscopic (EIS) study of pure TiO_2_ and ZnS‐doped nanocomposites.

By using cyclic voltammetry, the electrochemical behavior of pure TiO_2_ and ZnS‐doped nanocomposites was investigated over a potential range of −2V to +2 V and a current range of −60 to 40 μA at a scan rate of 10 mV/s. On Nyquist plots, both ZnS nanocomposite‐modified electrodes and electrodes modified with TiO_2_ showed semicircles as shown in Figure 8a,b. The equivalent circuit consisted of *electron*‐transfer resistance (*R*_ct_), *solution resistance* (*R*_*s*_), and *double‐layer capacitance* (*C*_dl_). In the TiO_2_‐doped samples, capacitance was bigger, but semicircle diameters were smaller and charge transfer resistance was lower (Yang et al. 2023). Photoexcited electron–hole (e^−^/h^+^) pairs were effectively separated by the decrease in *R*_ct_ value (Bai et al. 2018). It was found that heterojunctions formed between TiO_2_ and ZnS facilitated this separation and movement of charge carriers, which reduced recombination and improved photocatalytic performance in TiO_2_‐doped ZnS nanocomposites as shown in Figure 8a,b. Due to the TiO_2_‐doped ZnS heterojunction, photoexcited holes migrated towards the interface, whereas electrons migrated toward the bulk. Based on the cyclic voltammetry data, the specific capacitance of the synthesized materials was calculated.

(7) $$C=\frac{I∆T}{m∆V}$$

where “*C*” is the specific capacitance in Fg^−1^, “*I*” is the galvanostatic discharge current in ampere, discharge time in seconds, “*V*” is the voltage range, and “*m*” is the mass of the active material in grams. The specific capacitance values of pure and ZnS‐doped nanocomposites were 144.91 and 120.11 F/g.

### 3.7 Photocatalytic Activity

**FIGURE 9:**
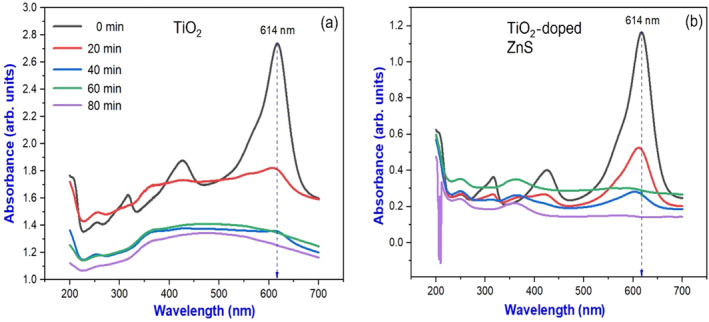
UV–Vis spectrum of MG dye degradation at different time intervals for (a) pure TiO_2_ and (b) TiO_2_‐doped ZnS nanocomposites.

**FIGURE 10:**
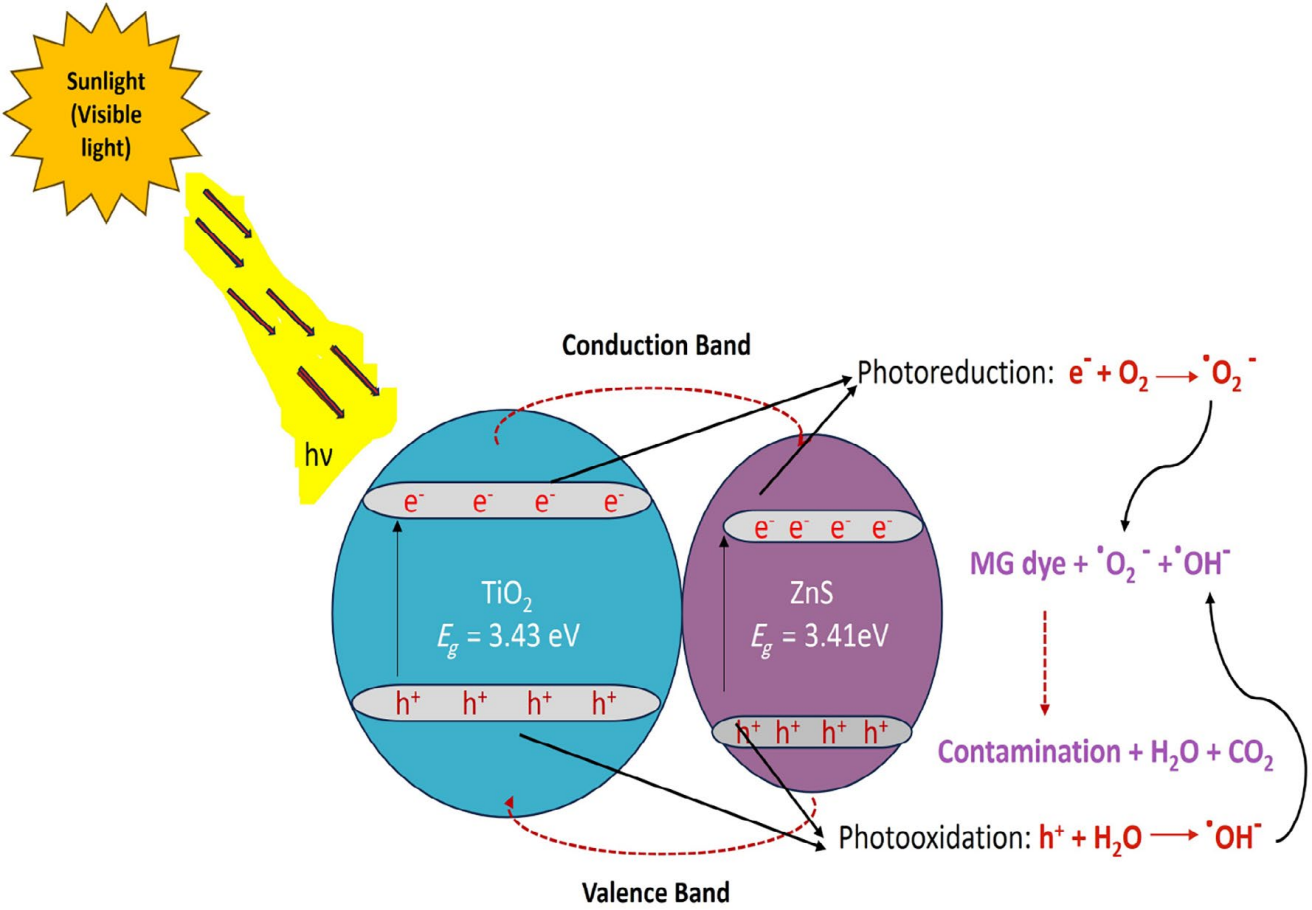
Photocatalytic mechanism of TiO_2_‐doped ZnS nanocomposites under visible light radiation.

An investigation of sunlight‐driven photocatalysis for the degradation of MG dye was presented in this study. In addition to pure TiO_2_ nanoparticles, ZnS nanocomposite doped with TiO_2_ served as catalysts. We prepared a stock solution that contained 20 mg/L of MG dye and separately added 0.3 g of either catalyst. To initiate the photocatalytic degradation process, 250 mL of the stock solution was transferred to a 500‐mL beaker and placed in direct sunlight. A UV–Vis spectrophotometer monitored the degradation of dye at 20‐min intervals for 80 min after the samples were exposed to sunlight. By doing so, it was possible to calculate the percentage of dye degradation over time. As illustrated in Figure 9a,b, the ultraviolet spectrum depicts the degradation of MG dye over time. As the dye degrades, there is a first‐order kinetics involved. A UV–Vis spectrum was analyzed at different time points to determine whether pure and TiO_2_‐doped ZnS nanocomposites had different absorption values. Maximum absorption was observed at 614 nm at the initial time point (*t* = 0). Due to the presence of the dopant, there is a slight shift in the maximum wavelength (Reddy et al. 2019; Djebbari et al. 2021). Under visible light exposure, Figure 10 shows a photocatalytic mechanism for degrading MG dye using pure TiO_2_ and TiO_2_‐doped ZnS nanocomposites.

The percentage efficiency of degradation of dye (Reddy et al. 2019; Dobrzanski et al. 2016) is given by Equation (8),

(8) $$\%D=\frac{A_{o}-A_{t}}{A_{o}\;}\times 100$$

**FIGURE 11:**
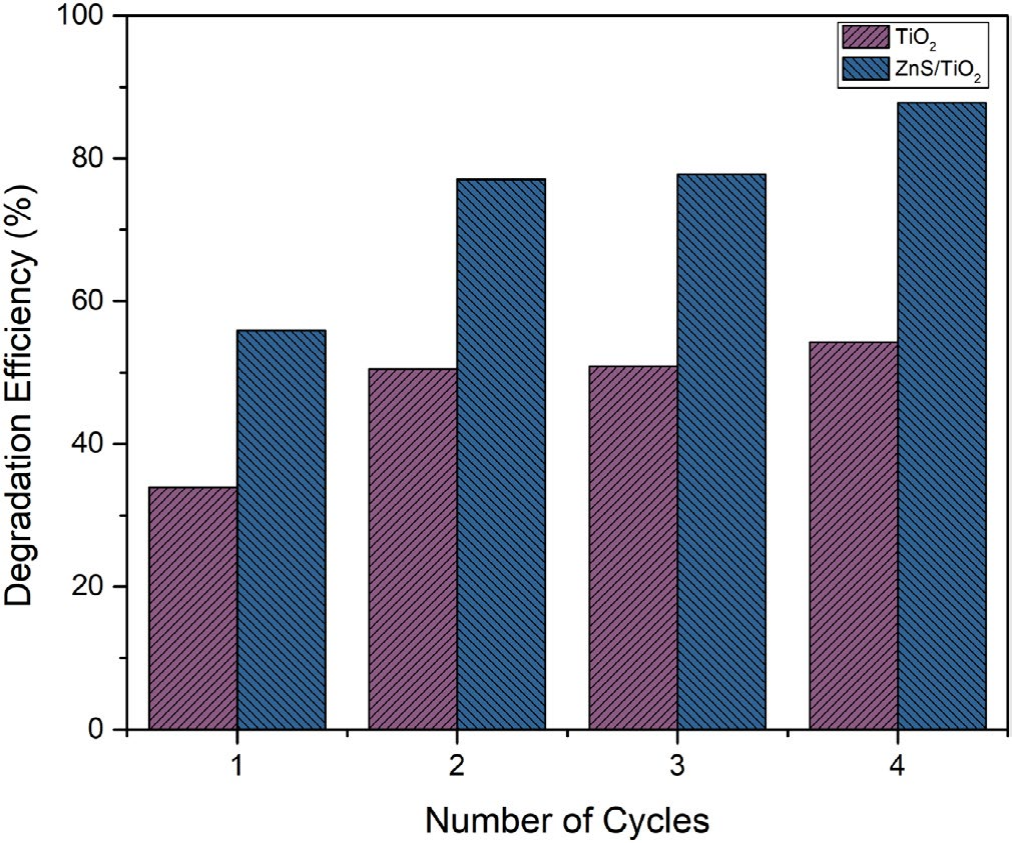
Degradation efficiency (%) of pure and TiO_2_‐doped ZnS nanocomposite.

We calculated the initial and final absorbance of the dye at the time *t* = 0 and 80 min, where *A*_*o*_ and *A*_*t*_ were the initial and final absorbance values. As can be seen in Figure 11, degradation has occurred.

Multiple cycles of 20‐min intervals were conducted between 0 and 80 min to evaluate the effectiveness of MG dye with two different types of nanoparticles. In comparison to pure nanoparticles, TiO_2_‐doped ZnS nanoparticles displayed a higher efficiency of 87.8%. From UV–Vis spectra, a specific relationship was obtained for the photocatalytic activity rate constant. In line with *Beer–Lambert*'*s principle*, the absorption has a direct relationship with concentration, making it possible to calculate the rate constant from the absorption values (Hensel et al. 2010; Bojinovaa et al. 2008).

(9) $$\ln\left(\frac{C_{0}}{C_{t}}\right)=\mathrm{kt}$$

**FIGURE 12:**
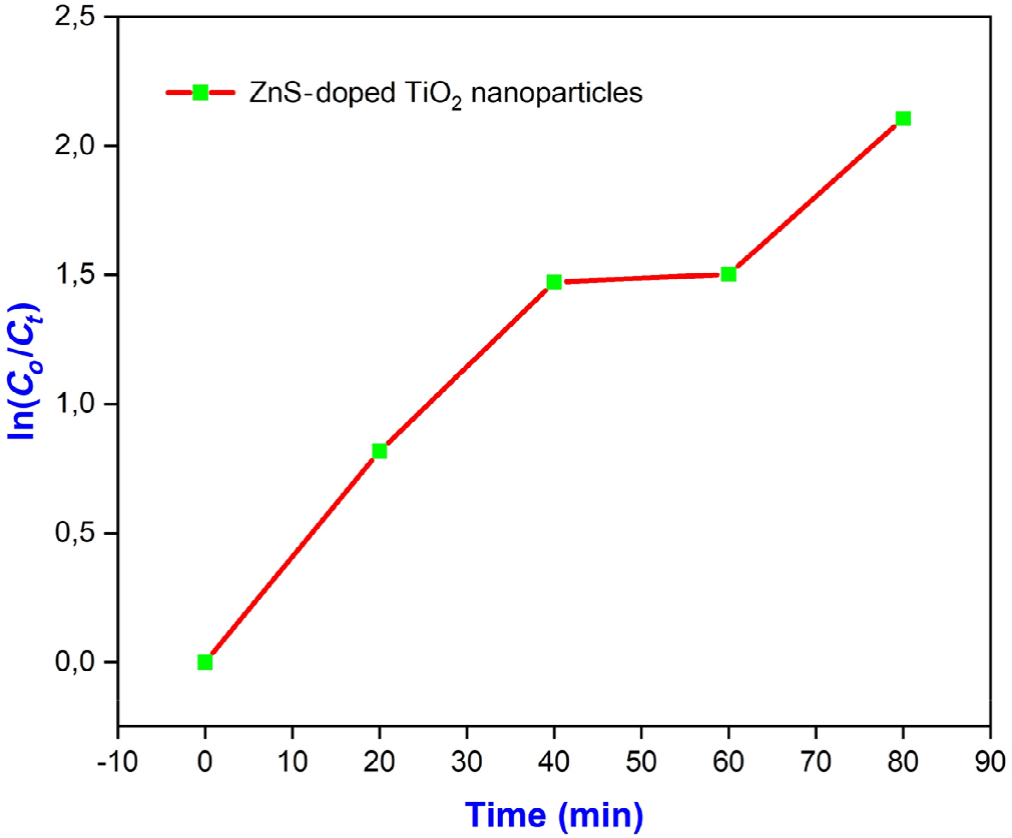
Apparent rate constant for the degradation of MG dye of pure TiO_2_ and TiO_2_‐doped ZnS nanocomposites.

When the ratio of initial concentration to concentration at a specific time, plotted against time, is analyzed, the slope of the natural logarithm of the ratio can be found, denoted by “*k*.” Using Figure 12, we can see the rate constant, *k*, for the photodegradation process. Photodegradation with sunlight exhibits *pseudo‐first‐order* kinetics (Sugimoto, Zhou, and Muramatsu 2002; Orudzhev et al. 2020; Chandrasekar et al. 2023) due to the nonlinear nature of the graph. Degradation rate constants for MG dye using pure and TiO_2_‐doped ZnS nanoparticles were 0.00931 and 0.02448 min^−1^, respectively. Doped nanoparticles degrade more rapidly than pure samples. When TiO_2_‐doped ZnS, the nanocomposites' degradation percentage is 87.8%, whereas the degradation percentage for pure TiO_2_ is 54% (Orudzhev et al. 2021; Gunasekaran et al. 2023), which shows that dopant enhances photocatalytic activity.

## 4 Conclusion

In comparison with the TiO_2_‐doped ZnS nanocomposites, the pure TiO_2_ nanoparticles had a crystallite size of 33 nm. By analyzing the XRD spectra, it was clear that the synthesized TiO_2_ nanoparticles were in the anatase phase. Nanoparticles doped with ZnS and pure TiO_2_ had an average particle size of 134 and 146 nm, respectively, which was determined by SEM. The elemental composition of the nanoparticles was determined by EDS. In pure TiO_2_ nanoparticles, Ti and O molecules were present, and Zn, S, Ti, and O molecules were present in TiO_2_‐doped ZnS nanocomposites. Specific wavelengths were observed for the vibrational, bending, and stretching modes of Ti—O—Ti. Both pure and doped TiO_2_ nanoparticles were found to be in the anatase phase based on their bandgap energy values from UV–Vis and PL spectra, and they are semiconductors in nature. These nanoparticles emit blue and violet lights in the 410–490 nm range. Measurements of the nanoparticles' specific capacitance were conducted electrochemically. For pure nanoparticles, degradation efficiency was ~54%, while degradation efficiency was ~87.8% for doped nanocomposites.

## Author Contributions

**S. Synthiya:** investigation, writing – original draft, visualization, methodology, formal analysis, data curation, resources, software. **T. Thilagavathi:** conceptualization, investigation, methodology, formal analysis, software, supervision, data curation, writing – review and editing, visualization, project administration. **R. Uthrakumar:** resources, formal analysis, software, investigation, conceptualization, visualization, methodology, project administration, data curation, supervision. **R. Renuka:** investigation, validation, methodology, formal analysis, data curation, resources, visualization, writing – review and editing. **C. Inmozhi:** conceptualization, investigation, validation, visualization, formal analysis, data curation, software, methodology, resources, writing – original draft, writing – review and editing. **K. Kaviyarasu:** funding acquisition, visualization, validation, formal analysis, project administration, software, resources, writing – review and editing.

## Ethics Statement

A component of the research process, whose purpose is to protect both the researcher and the participants in the research, which should have their dignity, rights, safety, and welfare respected.

## Conflicts of Interest

The authors declare no conflicts of interest.

## Data Availability

The datasets generated during and/or analyzed during the current study are available from the corresponding author on reasonable request.
